# Challenges of aging in the context of social inequalities

**DOI:** 10.11606/S1518-8787.201805200supl2ed

**Published:** 2018-10-25

**Authors:** Marilisa Berti de Azevedo Barros, Moisés Goldbaum

**Affiliations:** I Universidade Estadual de Campinas. Faculdade de Ciências Médicas. Departamento de Saúde Coletiva. Campinas, SP, Brasil; II Universidade de São Paulo. Faculdade de Medicina. Departamento de Medicina Preventiva. São Paulo, SP, Brasil

Population aging, which in the last century has gained increasing visibility as it spreads to different regions of the planet and due to the accelerated pace with which it has also advanced in countries of lesser economic development, is becoming one of the most challenging phenomena in this century because of its multiple consequences. With a growth rate of 3% a year, the demographic segment of 60 years of age and older currently represents 12.3% of the world’s population, and it is estimated that this percentage will rise to 21.3% by 2050. An even higher estimate exists for Brazil, with the prediction that by 2050, the older population will comprise 29.6% of the Brazilian population^1^.

As a result of the marked decline in fertility and mortality rates, population aging is a major achievement for mankind, but the new demographic and epidemiological scenario is now requiring new perspectives, conceptions, policies, technologies and models of care that enable healthy aging^2^.

To face this challenge, a number of initiatives have been developed, such as the World Assembly on Aging, by the United Nations, and the launching of the Active Aging Plan and the Global Guide: Age-Friendly Cities, by the World Health Organization^3^ ^,^ ^4^. In Brazil, following the trend of international movements, the National Aging Policy^5^ and the National Health Policy for the Elderly^6^ were established in the 1990s, to promote active and healthy aging. This process is reinforced by the promulgation of the Statute of the Elderly^7^ and by active aging^8^ commitments formalized by the Brazilian government.

In addition to initiatives by numerous organizations, the aging process has provoked intense scientific research and conceptual elaboration to support the understanding of this phenomenon and the monitoring of its social and epidemiological profile. Conceptual frameworks in this field have sought to broaden the scope beyond disease prevention and patient care, to include strategies that create opportunities for older population segments to participate in economic, social, cultural, intellectual, physical, civic and political activities^3^.

A document launched by the International Center for Longevity in Brazil in 2015 reinforces initiatives related to the four pillars of active aging - health, lifelong learning, participation and security - and points to the need to consider the implications of converging global trends, such as urbanization, globalization, migration, technological revolution, environmental and climatic changes, poverty and inequalities and the evolution of human rights^9^.

Regarding this, the implications of social inequality on the health of the older adults, as well as on the health of the population in general, are even more important in the face of the fact that income and wealth concentration has increased worldwide. Data from several countries analyzed by Piketty^10^ revealed that, after a significant decline observed from 1910-1920 to 1970-1980, income inequality increased again, reaching in 2010 the same levels as in 1910-1920. Oxfam’s international report draws attention to the extreme inequality that was reached in 2015, revealing that the richest 1% of the world’s population accumulates more wealth than the rest of the world^11^. More recent information on the situation in Brazil indicates that the country ranks 10^th^ in the world in terms of income concentration, but is the 1^st^ in relation to the degree of income concentration in the wealthiest percentile of the population^12^. Research shows that increases in income concentration tend to be accompanied by social inequality increases in mortality rates and other health indicators^13–15^.

The occurrence of an economic crisis increases the importance of analyzing social inequality in the health of the older adults. Research in several European countries about the impact of the economic crisis, which began in 2008, found that the austerity measures imposed were characterized by deep cuts in spending, especially in the public services of education, health and social security, by privatization of public services and deregulation of the market, which was followed by unemployment, flexibilization of labor contracts, wage and benefits cuts, with widespread dismantling of the public sector^16–18^. Pujolar et al.^18^ conclude that these policies conducted under a neoliberal model “have only contributed to the erosion of the mechanisms that reduce inequality and that enable the growth of equity”, a view that is being shared by the proponents of austerity policies, such as the International Monetary Fund, who began to recognize that the measures implemented did not lead to the expected results and undermined economic growth and equity^19^.

Brazil is recognized as one of the countries with the highest income concentration in the world. In addition, the country is undergoing an economic crisis and some austerity policies that have been implemented are similar to those applied in European countries. This scenario highlights the importance of analyzes of social inequalities in the health of the older adults, as has been done in several articles of this supplement. The articles also highlight the relevance and opportunity of monitoring the degree of inequalities in the follow-up of the older adults cohort that the ELSI-Brazil project can provide.

Analyzes on the most varied aspects of the health of the older adults have been developed in Brazil and many of them in population-based studies. Some research relies on population-based health surveys conducted in specific municipalities or regions. Several studies on the health of the older adults could be done nationally using data from the Vigitel, which cover populations living in Brazilian capitals and in the federal district with information obtained through telephone interviews. Other studies used data from National Surveys by Household Samples (PNADS), which allowed the creation of estimates for the Brazilian population as a whole. More recently, data from the first Brazilian National Health Survey (PNS), carried out in 2013, allowed many analyzes in relation to the Brazilian older adults as well. The first National Research on the Access and Use of Medicines (PNAUM), also in 2013, provided the evaluation of access and the profile of the use of medicines on a national scope.

However, the monitoring of the life and health conditions of the older population in a cohort of Brazilian older adults first took place with the development of the ELSI-Brazil project. The wide scope of issues in this project makes it possible to analyze the most diverse living and health conditions of the older adults. The analysis potential of the ELSI-Brazil project can be appreciated by all the articles presented in this supplement, which are dedicated to the analysis of data obtained from the cohort in the first stage of the research.
